# ENMD-1068 inhibits liver fibrosis through attenuation of TGF-β1/Smad2/3 signaling in mice

**DOI:** 10.1038/s41598-017-05190-7

**Published:** 2017-07-14

**Authors:** Quan Sun, Yan Wang, Jie Zhang, Jing Lu

**Affiliations:** Department of Laboratory Animal Science, School of Basic Medical Science, Capital Medical University, Beijing, 100069 China

**Keywords:** Extracellular signalling molecules, Liver fibrosis

## Abstract

Protease-activated receptor 2 (PAR-2) plays an important role in the pathogenesis of liver fibrosis. We studied the effect of N1-3-methylbutyryl-N4-6-aminohexanoyl-piperazine (ENMD-1068), a PAR-2 antagonist, on the development of CCl_4_-induced liver fibrosis in mice and activation of hepatic stellate cells (HSCs) isolated from the mice. Before CCl_4_ injection, the mice were injected intraperitoneally with either 25 mg/kg or 50 mg/kg ENMD-1068 or with 200 μL of the vehicle control twice per week for 4 weeks. The isolated HSCs were stimulated by TGF-β1 with or without ENMD-1068 to evaluate the role of PAR-2 in TGF-β1 induced HSCs activation and collagen production. We showed that the levels of ALT/AST, collagen content, and α-smooth muscle actin (α-SMA) were significantly reduced by treatment with ENMD-1068 in CCl_4_-induced fibrotic mice. Interestingly, we found TGF-β1 signaling-related expression levels of α-SMA, type I and III collagen, and C-terminal phosphorylation of Smad2/3 were significantly decreased in the ENMD-1068 treated HSCs. Moreover, we showed ENMD-1068 treatment inhibited trypsin or SLIGRL-NH_2_ stimulated calcium release and TGF-β1 induced Smad transcriptional activity in HSCs. We demonstrated that ENMD-1068 reduces HSCs activation and collagen expression through the inhibiton of TGF-β1/Smad signal transduction.

## Introduction

Liver fibrosis is the common pathological process in acute and chronic liver injury from a variety of causes and may progress to portal hypertension, hepatocellular carcinoma, and liver failure. A substantial body of evidence has confirmed hepatic stellate cells (HSCs) as the principal source of collagen produced during liver fibrosis, and there is considerable interest in factors that regulate HSCs activation and collagen expression^1, 2^. Despite the increasing understanding of liver fibrosis, treatment of these diseases remains limited because no efficient and well-tolerated drugs are available. The only treatment option currently available for severe end-stage liver disease is orthotopic liver transplantation. Therefore, there is a clear need to identify possible targets for therapeutic interventions. Among those, protease-activated receptors (PARs) are key candidates as they play a central role in inflammatory and fibrotic responses^3, 4^.

PARs are integral membrane proteins that are coupled to G-proteins. Unlike other G Protein-coupled Receptors (GPCRs), which are activated by ligand binding, PARs are irreversibly activated by proteolytic cleavage. PAR-1 was initially identified in the search for the cellular thrombin receptor. To date, four PARs have been identified. Thrombin activates PAR-1, 3, and 4, and factor Xa activates PAR-1 and PAR-2. PAR-2 is also activated by trypsin, mast cell tryptase, and the tissue factor/factor VIIa and factor Xa complex^5^.

Importantly, activated PAR-2 mediates several pathophysiological procedures involved in acute and chronic inflammatory and fibrotic diseases of the joints, skin, brain, lung, and gastrointestinal tract^6^. There is increasing evidence that PAR-2 is a critical contributor in the pathogenesis of liver fibrosis. Increased PAR-2 expression has been detected as rat stellate cells transformed to a myofibroblastic phenotype, and PAR-2 agonist tryptase and the peptide SLIGRL induced HSCs proliferation and collagen secretion^7^. In line with this finding, the prototypical PAR-2 agonist SLIGKV stimulates the proliferation of human HSCs and TGF-β1 production in a PAR-2 dependent manner^8^. Moreover, PAR-2 activation has been shown to be highly relevant to the progression of liver fibrosis in animal models. Rat HSCs express PAR-2 under normal conditions and is markedly increased in liver fibrosis^4^. It has been shown that genetic ablation of PAR-2 in mice affords protection from liver fibrosis, as evident from a reduction in the extent and severity of fibrotic lesions and diminished collagen expression^8^. In addition, treatment of rats with liver fibrosis with tryptase inhibitor APC366 results in amelioration of collagen production through the involvement of PAR-2^9^.

There is an overwhelming amount of data proving that PAR-2 plays an important role in liver fibrosis. PAR-2 antagonists have recently been developed and may represent a novel therapeutic approach in preventing fibrosis in patients with chronic liver disease. N1-3-methylbutyryl-N4-6-aminohexanoyl-piperazine (ENMD-1068) is a novel selective antagonist of PAR-2 and can prevent PAR-2 activation from genomic intervention^10, 11^. In the present study, a carbon tetrachloride (CCl_4_) induced mouse model of liver fibrosis was used to assess the effect of ENMD-1068 on the development of liver fibrosis. The effect of ENMD-1068 treatment in the mouse model was evaluated by collagen production, α-SMA expression, and alanine aminotransferase/aspartate aminotransferase (ALT/AST) level. In addition, HSCs isolated from mice livers were used to analyze the effect of ENMD-1068 treatment in the activation and collagen release of HSCs.

## Methods

### Mouse models

The study protocol was approved by the Animal Experiments and Experimental Animal Welfare Committee of Capital Medical University (Permit Number: AEEI-2016–150). ICR mice used in this study were purchased from Beijing Vital Laboratory Animal Technology Co. (Beijing, China).

**Figure 1:**
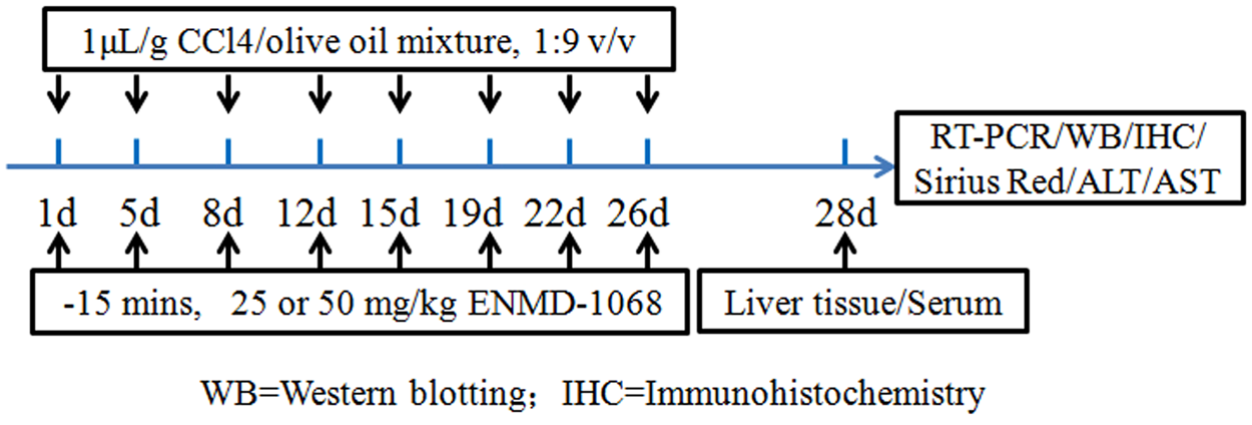
Time schedule for CCl_4_ and ENMD-1068 treatment.

Mouse model of liver fibrosis was induced by injection of CCl_4_. Eight-week-old adult ICR mice received intraperitoneal injections of 1 μL/g body weight of a CCl_4_/olive oil (OO) mixture, 1:9 v/v, twice per week. Control mice were treated with 1 μL/g body weight of OO. Mice were sacrificed at 4 weeks after CCl_4_ treatment, on the day after the last injection. 25 mg/kg or 50 mg/kg ENMD-1068 (Abcam, Shanghai, China) with 200 μL of the vehicle were administered intraperitoneally 15 mins before CCl_4_ treatment twice per week for 4 weeks (N = 6 per group). As shown in Figs 1, 4 weeks after CCl_4_ treatment, the animals were sacrificed by CO_2_ exposure and liver tissues were collected for Sirius Red staining, immunohistochemistry staining, RT-PCT and Western blotting. Serum was isolated for analyzing the serum levels of ALT and AST. All animal work was performed under the guidelines of the Ethics Committee of Capital Medical University.

### Isolation and culture of primary mouse HSCs

Primary mouse HSCs were isolated from adult male ICR mice by collagenase perfusion and purified by density gradient in Nicodenz (AXIS-SHIELD PoC, Scotland). All cells were maintained in Dulbecco’s modified Eagle’s medium (DMEM) supplemented with 10% fetal bovine serum (FBS). To evaluate the role of PAR-2 antagonist on HSCs activation and collagen induction, HSCs were stimulated with TGF-β1 (5 ng/mL, Abcam, Shanghai, China) for 24 h in the presence or absence of the selective inhibitor of PAR-2 (ENMD-1068, 10 mM). HSCs were serum starved for 24 h before inhibitor incubation.

### Serum biochemical parameters

On day 28 after CCl_4_ injection, serum was collected by orbital venous plexus blood collection. The blood serum was separated, and the levels of AST and ALT were measured by standard enzymatic assay kits. Each assay is a colorimetric assay with detection of a highly-colored end product measured at 490–520 nm by a spectrophotometer (Hitachi 736–10, Beijing, China). The absorbance of each end product is proportional to the enzyme’s activity.

### Quantitative analysis of liver fibrosis

Liver sections were stained with Sirius Red for collagen visualization. In brief, liver tissues were fixed in PBS containing 4% paraformaldehyde for 24 h and embedded in paraffin. Four micron-thick sections from paraffin-embedded liver tissue were deparaffinized and stained with Sirius Red solution for 1 h. The fibrotic area was assessed by computer-assisted image analysis with Leica Qwin V3 software (Lecia Microsystems, Heidelberg, Germany). The mean value of 15 randomly selected areas per sample was used to estimate the percentage of fibrotic area.

### Real-time RT-PCR

Total RNA was extracted from liver tissue using the Total RNA Kit (R6834, Omega, GA, USA) and analyzed by quantitative PCR using SYBR Green qPCR Master Mix (1206352, Applied Biosystems, Warrington, UK) on an ABI 7500 System (Applied Biosystems, NY, USA). Primers were as follows: 18 S rRNA: sense, 5′-GTA ACC CGT TGA ACC CCA TT-3′; antisense, 5′-CCA TCC AAT CGG TAG TAG CG-3′. Mouse a-SMA: sense, 5′-ATG CTC CCA GGG CTG TTT T-3′; antisense, 5′-TTC CAA CCA TTA CTC CCT GAT GT-3′. Mouse Col α1(I):sense, 5′-AGG GCG AGT GCT GTG CTT T-3′; antisense, 5′-CCC TCG ACT CCT ACA TCT TCT GA-3′. Mouse Col α1(III): sense, 5′-TGA AAC CCC AGC AAA ACA AAA-3′; antisense, 5′-TCA CTT GCA CTG GTT GAT AAG ATT AA-3′.

### Immunohistochemical staining

Immunohistochemical staining for α-SMA (Cat. No.A5228, 1:100, Sigma-Alorich), C-terminal phospho-Smad2 (Cat. No.ab53100, 1:100, Abcam) and C-terminal phospho-Smad3 (Cat. No.ab52903, 1:100, Abcam) were carried out using immunohistochemistry kits (Boster Biological Engineering Co., Wuhan, China) per the manufacturer’s instructions. The yellow-stained areas in the sections were assessed with an image analyzer (Image-Pro Plus, MediaCybernetics, Rockville, MD, USA) for semi-quantitative analysis. The results were expressed as the area density (area of the positive cells/area of the whole field).

### Measurement of calcium mobilization

Primary mouse HSCs were washed and incubated in the dark with 3 mM Oregon green BAPTA-1 AM(Thermo Fisher, Beijing, China) in the presence of 1% pluronic acid for 45 min at 37 °C. The excess of fluorochrome was removed by two washing steps. HSCs were then resuspended at densities of 10^4^ cells per well in DMEM medium and incubated with 10 mM ENMD-1068 or with an equivalent volume of dimethylsulfoxide (DMSO) before inducing internal calcium mobilization with 0.2 U/mL trypsin, 10 μΜ SLIGRL-NH_2_ or 10 μΜ TFLLR-NH_2_ (PAR1 agonist). Changes in fluorescence were recorded as previously described^12^.

### Transient transfections and reporter gene assays

Primary mouse HSCs received Lipofectamine 2000 (Life Technologies) with TGF-β1/Smad responsive reporter genes REPO^TM^SMAD (Genomeditech Co., shanghai, China) as previous described^13^. Transfection efficiency was normalized by cotransfection of Renilla luciferase reporter plasmid pRL-TK (Promega, Madison, WI). 24 h after the transfection, cells were treated with TGF-β1(5 ng/mL) or ENMD-1068 (10 mM) for 24 h. The data were derived from three wells processed in parallel and normalised with Renilla luciferase activity.

### Immunoblotting

Proteins isolated from mouse HSCs were denatured and separated by electrophoresis on SDS-polyacrylamide gels followed by protein transfer to nitrocellulose membranes. Membranes were blocked at room temperature for 1 h in 5% milk proteins in PBS containing 0.01% Tween 20 (PBST). Primary antibodies in PBST were applied to the membrane and incubated at 4 °C overnight. Primary antibodies are as follows: anti-mouse α-SMA (Cat. No.A5228, 1:1000, Sigma-Alorich), anti-mouse collagen І (Cat. No.ab6308, 1:200, Abcam), anti-mouse collagen III (Cat. No.ab7778, 1:200, Abcam) anti- C-terminal phospho-Smad2 (Cat. No.ab53100, 1:1000, Abcam),anti-C-terminal phospho-Smad3 (Cat. No.ab52903, 1:2000, Abcam) anti-Smad2 (Cat. No.ab33875, 1:1000, Abcam), anti-Smad3 (Cat. No.ab40854, 1:1000, Abcam), anti- β-Actin (Cat. No.12262, 1:1000, Cell Signaling). Goat anti-mouse IgG labeled with HRP (Cat. No.ab6789, 1:2000, Abcam) was used as secondary antibodies. Immunoreactive signals were detected using an Enhanced Chemiluminescence (ECL) kit (Amersham Pharmacia Biotech) through an ECL system. The results were quantified using Image J 1.43 (National Institutes of Health, Bethesda, MD) after densitometric scanning of the films. Western blot signals were normalized relative to the image of the appropriate control samples. Results of a minimum of three independent Western blot analyses were averaged and pooled to yield the data shown in the histograms.

### Immunofluorescence Imaging

After treated with TGF-β1 (5 ng/mL) or ENMD-1068 (10 mM) for 24 h, primary mouse HSCs grown on the coverslips were fixed in 4% paraformaldehyde and stained with anti-α-SMA (Cat. No.A5228, 1:100, Sigma-Alorich) and anti-p-Smad2 (Cat. No.ab33875, 1:100, Abcam) antibodies, followed by fluorescein isothiocyanate (FITC) and tetramethylrhodamine isothiocyanate conjugated secondary antibodies using standard procedures. Nuclei were stained with 4′,6-diamidino-2- phenylindole (DAPI, Cat. No.D9542, Sigma-Aldrich), followed by analysis with confocal microscopy (LSM510Meta, Zeiss, Germany).

### Statistical analysis

Data are presented as mean ± standard deviation (SD). The data between groups were analyzed for statistical differences using SPSS 17.0 statistical software (SPSS Institute, USA) and one-way analysis of variance tests plus subsequent Bonferroni post hoc test. The p-value was two-tailed and considered as statistically significant or highly significant if it was less than 0.05 or 0.01, respectively.

## Results

### Biochemical parameters

**Figure 2:**
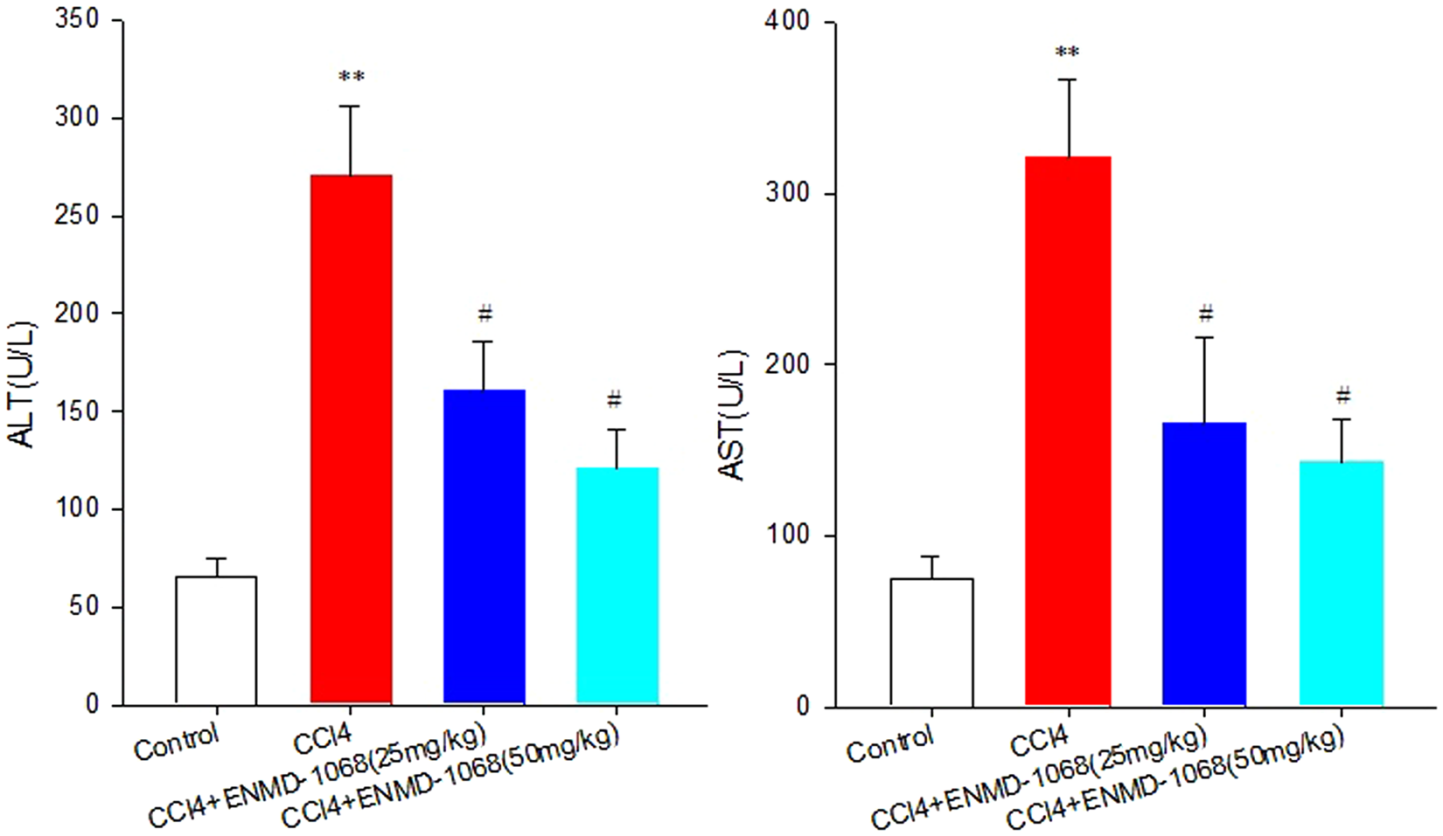
Effect of ENMD-1068 on ALT and AST levels. Control mice were treated with olive oil. Mice in CCl_4_ group received intraperitoneal injections of CCl_4_ twice per week. 25 mg/kg or 50 mg/kg ENMD-1068 were administered intraperitoneally 15 mins before CCl_4_ treatment twice per week for 4 weeks. All animals were sacrificed at 4 weeks after CCl_4_ treatment and serum was collected for ALT/AST test. Relative to control, ENMD-1068 treatment at both 25 and 50 mg/kg reduced ALT and AST levels. N = 6, bars represent SD, ^**^P < 0.01vs Control, ^#^P < 0.05 vs CCl_4_.

After CCl_4_ injection for 4 weeks, there was no mortality in any of the experimental groups. However, all the CCl_4_ treated mice showed progressive listlessness, reduced movement and increasing abdominal girth. Liver injury was assessed by determining the serum levels of liver enzymes including ALT and AST. As shown in Fig. 2, the serum level of ALT and AST were markedly increased in the CCl_4_-treated mice compared with the control mice (270.80 ± 35.6 vs 65.40 ± 9.76 P < 0.01 for ALT; 320.80 ± 45.60 vs 75.30 ± 12.27 P < 0.01 for AST). The increases were suppressed in both ENMD-1068 25 mg/kg (160.34. ± 26.36 vs 270.80 ± 35.6 P < 0.05 for ALT; 165.34 ± 50.30 vs 320.80 ± 45.60 P < 0.05 for AST) and ENMD-1068 50 mg/kg (120.90 ± 20.16 vs 270.80 ± 35.6 P < 0.05 for ALT; 143.25 ± 25.20 vs 320.80 ± 45.60 P < 0.05 for AST) treatment groups. There was no significant difference between ENMD-1068 50 mg/kg treatment and ENMD-1068 25 mg/kg treatment (P > 0.05). These results showed that ENMD-1068 treatment can alleviate liver injury induced by CCl_4_.

### Inhibition of liver fibrosis by ENMD-1068

**Figure 3:**
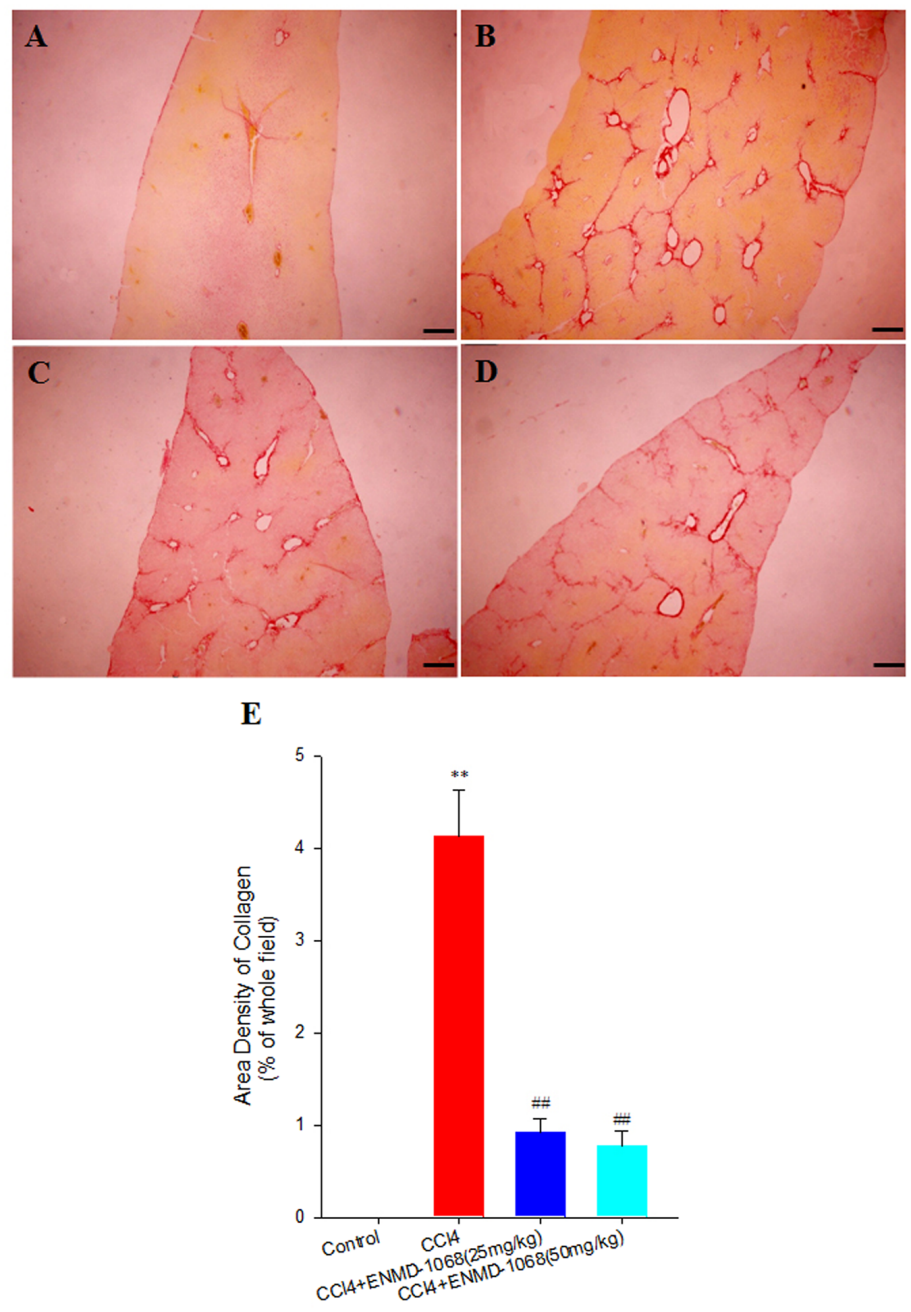
Sirius Red staining of collagen in liver sections. Control mice were treated with olive oil. Mice in CCl_4_ group received CCl_4_ twice per week. For ENMD-1068 treated mice, 25 mg/kg or 50 mg/kg ENMD-1068 were administered intraperitoneally 15 mins before CCl_4_ treatment twice per week for 4 weeks. All animals were sacrificed at 4 weeks after CCl_4_ treatment and liver tissues were collected for Sirius Red staining. (**A**–**D**) Representative images of Sirius Red staining for the (**A**) Control, (**B**) CCl_4_, (**B**) CCl_4_ + ENMD-1068 (25 mg/kg) and (**D**) CCl_4_ + ENMD-1068 (50 mg/kg). (**E**) Area density of collagen in representative images for each group; N = 6, bars represent SD, ^**^P < 0.01 vs Control, ^##^P < 0.01 vs CCl_4_, bar = 50 μm.

To evaluate the effect of ENMD-1068 on liver fibrosis, animals with intraperitoneal injection of CCl_4_ that had been allowed to establish liver fibrosis were treated with one of two doses of ENMD-1068 or with saline solution as a negative control. Four weeks later, liver tissues were collected for paraffin sectioning. As shown in Fig. 3, morphometric analysis of Sirius Red staining revealed that collagen was increased significantly in CCl_4_ group compared with the control group (4.13% ± 0.51% vs 0, P < 0.05). Collagen deposition was markedly attenuated in the high dose (50 mg/kg) ENMD-1068 group compared with the saline group (0.77% ± 0.16% vs 4.13% ± 0.51%, P < 0.01). Similar results were obtained after administration of the low dose (25 mg/kg) ENMD-1068 in CCl_4_-treated mice (0.92% ± 0.15% vs 4.13% ± 0.51%, P < 0.01). There was no significant difference between ENMD-1068 50 mg/kg treatment and ENMD-1068 25 mg/kg treatment (P > 0.05). These results demonstrated that liver fibrosis was attenuated by ENMD-1068 treatment.

**Figure 4:**
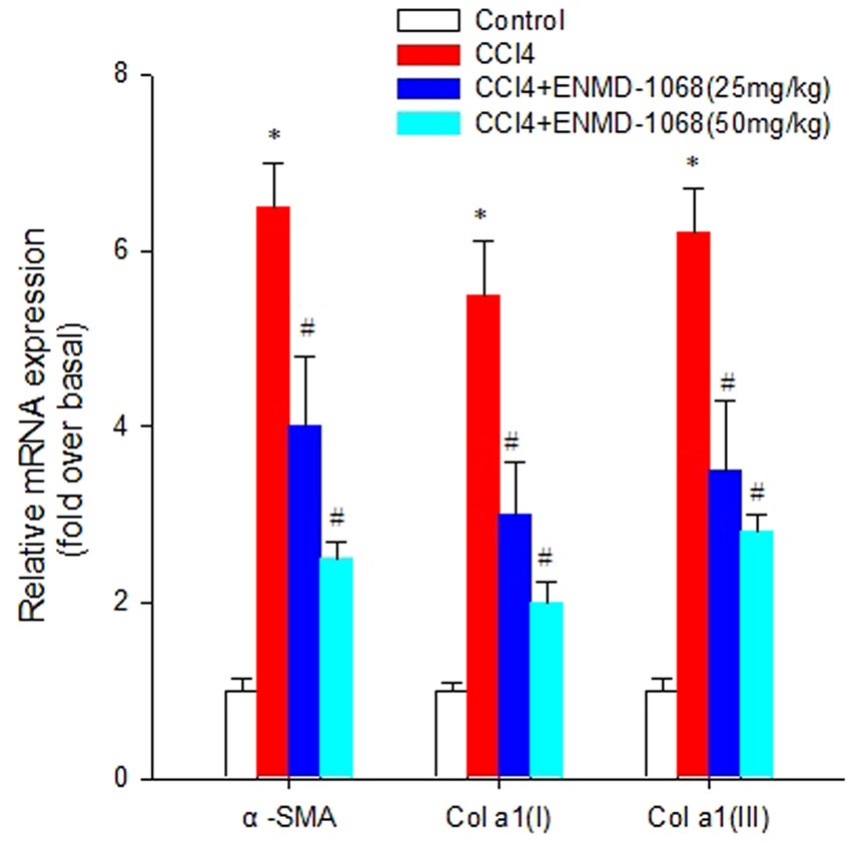
ENMD-1068 inhibits the mRNA expression of fibrotic marker in liver tissues. Mice were divided into 4 groups randomly. Control mice were treated with olive oil only. Mice in CCl_4_ group received CCl_4_ twice per week. In ENMD-treated group, mice were administered intraperitoneally 25 mg/kg or 50 mg/kg ENMD-1068 15 mins before CCl_4_ treatment twice per week for 4 weeks. All animals were sacrificed at 4 weeks after CCl_4_ treatment and liver tissues were co llected to investigate the effect of ENMD-1068 on the mRNA expression of fibrotic marker. The mRNA expression of fibrotic markers was quantified using real-time RT-PCR. 18 S rRNA was used for normalization of PCR data for the genes of α-SMA, Colα1(I) and Colα1(III). N = 6, bars represent SD, ^*^P < 0.05 vs Control, ^#^P < 0.05 vs CCl_4_.

To evaluate the effect of ENMD-1068 on HSC activation, we analyzed the mRNA levels of fibrotic marker in liver tissues, including α-SMA, Col α1(І) and Col α1(III), by real-time qPCR after ENMD-1068 administration for 4 weeks. We found that CCl_4_ treatment increased significantly the mRNA levels of α-SMA, Col α1(І) and Col α1(III) compared to the control mice, while ENMD-1068 administration resulted in the reverse effect (Fig. 4).

### Effect of ENMD-1068 on α-SMA, p-Smad2 and p-Smad3 expression

**Figure 5:**
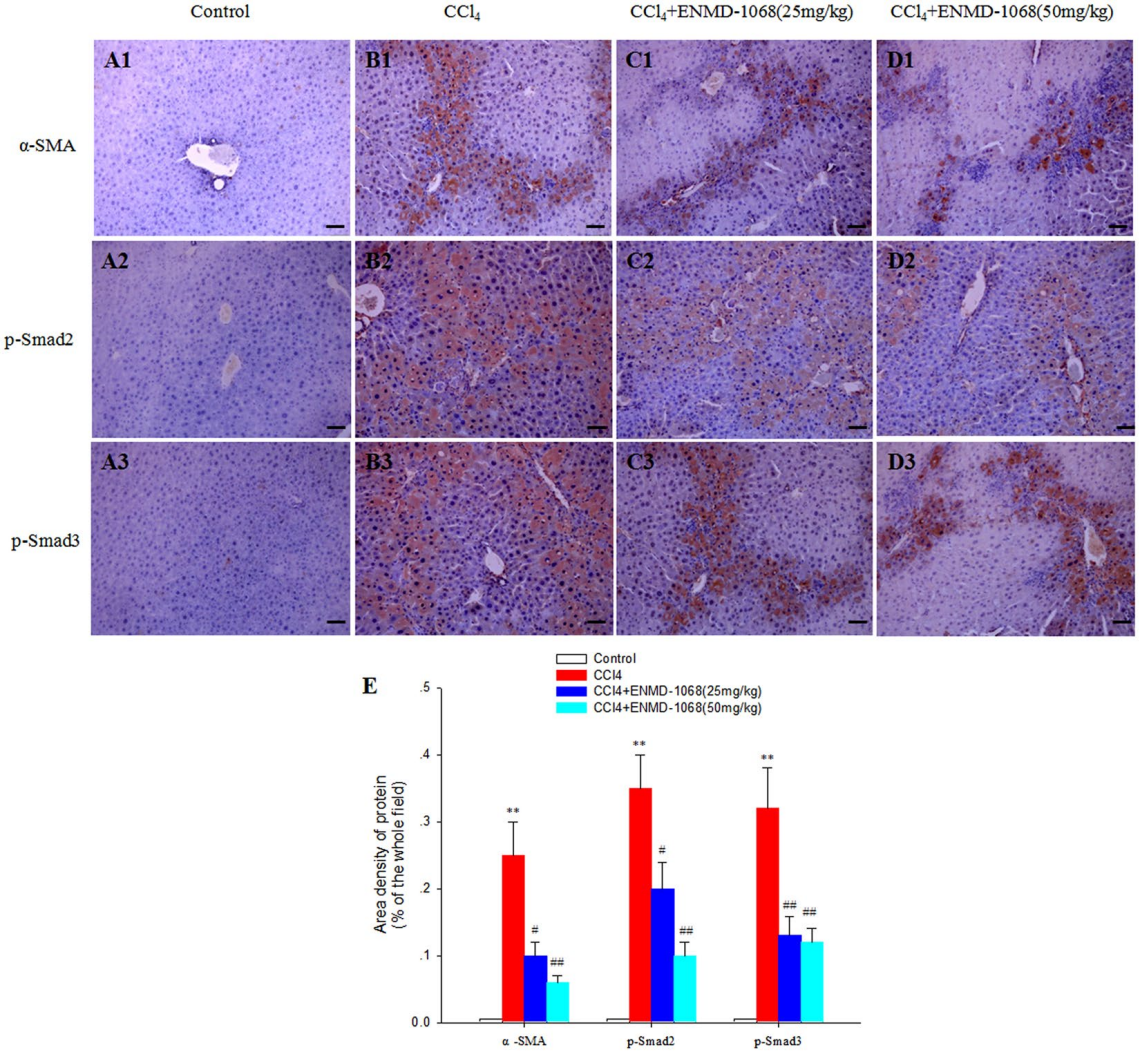
Effect of ENMD-1068 on α-SMA and p-Smad2/3 expression in liver sections. α-SMA and p-Smad2/3 immunohistochemistry were performed to measure effect of ENMD-1068 on HSCs activation in liver sections in (**A1–3**) Control, (**B1–3**) CCl_4_, (**C1–3**) CCl_4_ + ENMD-1068 (25 mg/kg) and (**D1–3**) CCl_4_ + ENMD-1068 (50 mg/kg) mice. (**E**) Relative to CCl_4_ treatment, ENMD-1068 treatment at both 25 and 50 mg/kg reduced significantly the expression of α-SMA and p-Smad2/3. N = 6, bars represent SD, ^**^P < 0.01 vs Control, ^##^P < 0.01, ^#^P < 0.05 vs CCl_4_, bar = 50 μm.

To assess whether ENMD-1068 is effective in inhibiting activation of HSCs *in vivo*, α-SMA, p-Smad2 and p-Smad3 immunohistochemistry were performed with all liver sections. There were minimal α-SMA, p-Smad2 and p-Smad3 immunostaining detected in the control mice (Fig. 5A1–3). By contrast, immunohistochemical staining for α-SMA, p-Smad2 and p-Smad3 were positive in the CCl_4_ group. Very strong α-SMA, p-Smad2 and p-Smad3 staining were observed mainly in the area of collagen production (Fig. 5B1–3), which indicates that the increased expression of α-SMA, p-Smad2 and p-Smad3 relate to collagen production. As shown in Fig. 5E, the content of α-SMA, p-Smad2 and p-Smad3 positives in the CCl_4_ group were significantly higher than that in the control and ENMD-1068 group (P < 0.01). Compared with the control vehicle treatment, treatment with ENMD-1068 25 mg/kg and ENMD-1068 50 mg/kg significantly decreased the expression of α-SMA, p-Smad2 and p-Smad3 (Fig. 5C1–3 and Fig. 5D1–3, P < 0.05). There was no significant difference between ENMD-1068 50 mg/kg treatment and ENMD-1068 25 mg/kg treatment (P > 0.05).

### Effect of ENMD-1068 on intracellular calcium mobilization

**Figure 6:**
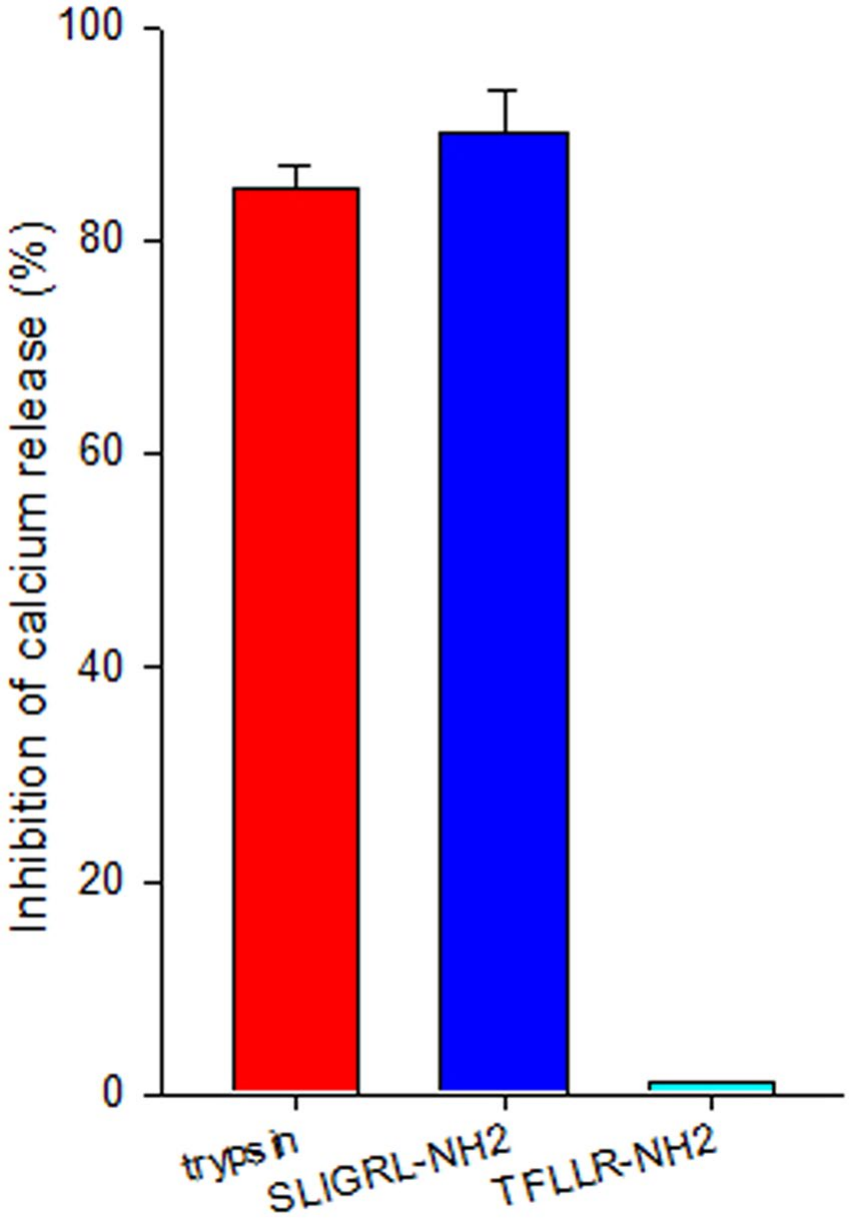
Effect of ENMD-1068 on trypsin or SLIGRL-NH_2_ induced [Ca2+]i mobilization. Oregon Green BAPTA-1 AM-loaded mouse HSCs were incubated with dimethylsulphoxide (DMSO) or ENMD-1068 (10 mM) and stimulated with 0.2 U/mL trypsin or 10 μΜ SLIGRL-NH_2_(PAR-2 agonist). Data represent mean percentage of inhibition compared with mean peak calcium concentration with DMSO. Red bar represents trypsin-activated HSCs. Blue bar indicates SLIGRL-NH_2_-stimulated HSCs. Cyan bar represents TFLLR- NH_2_-stimulated HSCs. N = 3.

We next investigated whether ENMD-1068 interferes with HSCs calcium mobilization after activation of PAR-2. As shown in Fig. 6, pretreatment of HSCs with 10 mM ENMD-1068 strongly inhibited trypsin or SLIGRL-NH_2_, but not TFLLR-NH_2_ (PAR1 agonist) induced [Ca^2+^]_i_ mobilization. Dimethylsulphoxide (DMSO) has no effect on trypsin, SLIGRL-NH_2_ and TFLLR-NH_2_ induced [Ca^2+^]_i_ mobilization in HSCs.

### ENMD-1068 blocks TGF-β1/Smad signaling in primary mouse HSCs

**Figure 7:**
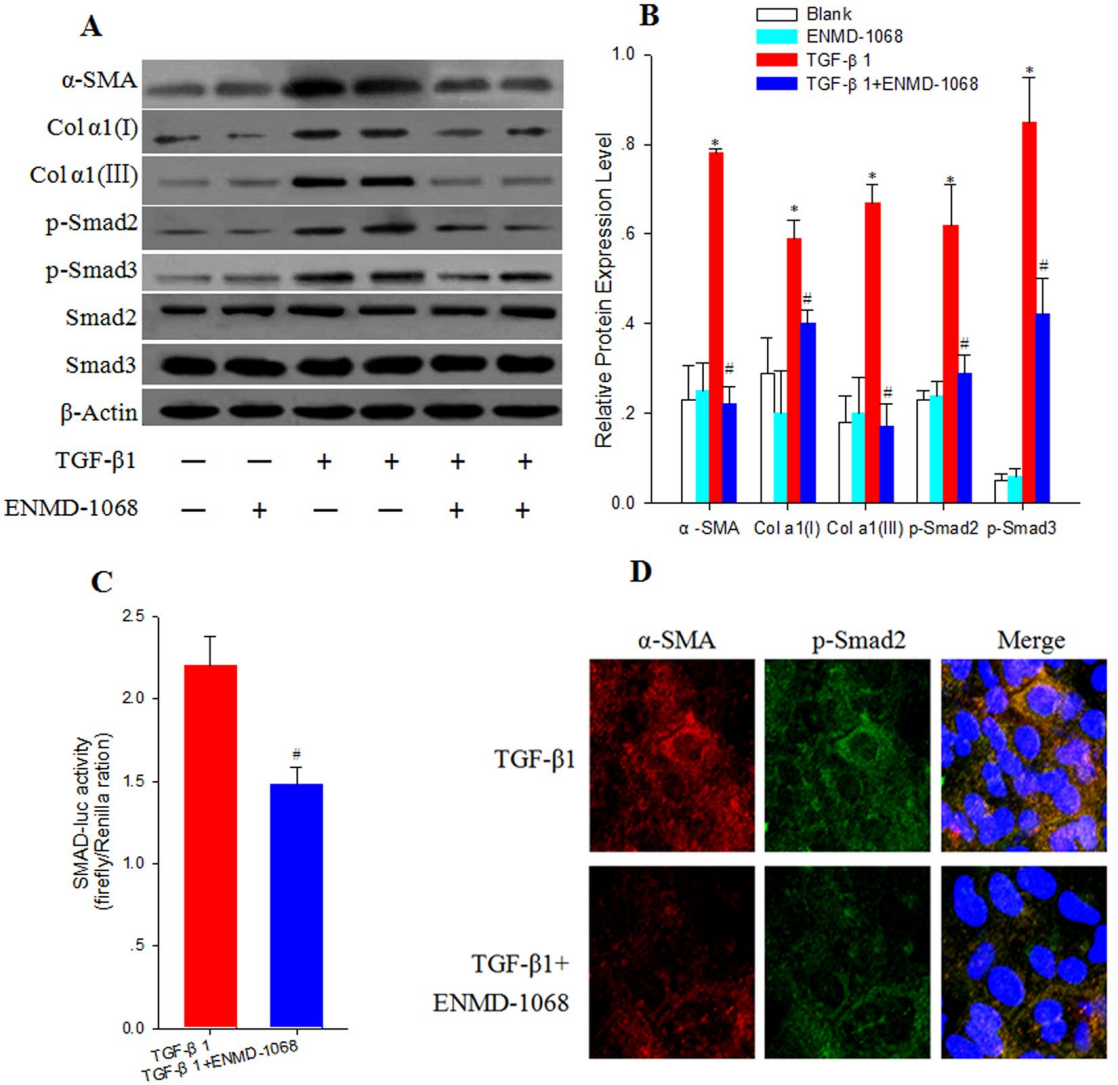
ENMD-1068 reduces HSCs activation and collagen production through the inhibition of TGF-β1/Smad signal transduction. (**A**) immunoblotting for α-SMA, Collagen I, Collagen III, and Smad2/3 C-terminal phosphorylation following stimulation with TGF-β1 (5 ng/mL) for 24 h in the presence or absence of the selective inhibitor of PAR-2 (ENMD-1068, 10 mM). (**B**) histogram showing densitometry analysis and quantification of α-SMA, Collagen I, Collagen III, and Smad2/3 C-terminal phosphorylation. The signals for α-SMA, Collagen I, Collagen III, p-Smad2C and p-Smad3C were normalized to respective bands for β-actin and/or Smad2/3. (**C**) Primary mouse HSCs were cotransfected with SMAD luciferase reporter plasmid and pRL-TK vector. Twenty-four hours after transfection, cells were treated with TGF-β1(5 ng/mL) in the presence or absence of ENMD-1068 (10 mM) for another 24 h. Luciferase activity was determined with the commercial luciferase reporter assay system. (**D**) Confocal microscopy images displayed the subcellular localization of α-SMA (red) and p-Smad2 (green) in merged image panels. The nuclei were stained with 4′,6-diamidino-2-phenylindole (DAPI; blue)(×200). (mean ± SD; ^*^P < 0.05 vs Blank; ^#^P < 0.05 vs TGF-β1 treatment, N = 3).

TGF-β1/Smad signal pathway plays a crucial role in HSCs activation and collagen production. To explore the role of PAR-2 in regulating TGF-β1/Smad signal, HSCs were stimulated with TGF-β1 (5 ng/mL) for 24 h in the presence or absence of the selective inhibitor of PAR-2 (ENMD-1068, 10 mM). As shown in Fig. 7A and B, α-SMA, Col α1(І),Col α1(III), and Smad2/3 C-terminal phosphorylation expression levels were increased significantly following stimulation with TGF-β1 compared with the no treatment HSCs (P < 0.05). The increases were suppressed in ENMD-1068 treated group compared with the TGF-β1 treated only group (P < 0.05). Luciferase assays showed that ENMD-1068 caused significant decrease in luciferase activity compared with TGF-β1 treated HSCs indicating that ENMD-1068 inhibited the Smad transcriptional activity in HSCs (P < 0.05, Fig. 7C). The colocalization of α-SMA and p-Smad2 in HSCs was examined by immunofluorescence imaging with confocal microscopy. As shown in Fig. 7D, α-SMA and p-Smad2 were observed predominantly in the cytoplasm. Furthermore, α-SMA exhibited excellent colocalization with p-Smad2. Colocalization of α-SMA and p-Smad2 were markedly increased after TGF-β1 stimulation for 24 h, which were reduced in ENMD-1068 treated HSCs. These results showed that ENMD-1068 reduced HSCs activation and Col α1(І) and Col α1(III) production through the inhibition of TGF-β1/Smad signal transduction.

## Discussion

Liver fibrosis is a common response to chronic liver injury, ultimately leading to cirrhosis and its complications including portal hypertension, liver failure, and hepatocellular carcinoma. Despite the relatively large population of patients suffering from hepatic fibrosis and cirrhosis, no efficient and well-tolerated drugs are available for the treatment of this disorder^14^. Increasing evidence suggests that PAR-2 plays an important role in HSCs activation and liver fibrosis, and PAR-2 antagonists may represent a novel therapeutic approach in preventing fibrosis in patients with chronic liver disease^4, 7–9, 15^. We aimed to evaluate the efficacy of PAR-2 antagonists in suppressing HSCs activation and progression of liver fibrosis. Our results confirmed that ENMD-1068, a protease-activated receptor 2 antagonist, contributes to a reduction in hepatic collagen content and histological fibrosis accompanied by decreased HSCs activation, as demonstrated by the reduced expression of α-SMA. Furthermore, we provide evidence *in vitro* that inhibition of PAR-2 reduced TGF-β1 induced phosphorylate Smad2 and expression of α-SMA and collagen.

Testing the efficacy of novel therapeutics in an *in vivo* animal model is a crucial step in a preclinical efficacy evaluation. According to the most widely accepted mechanism for liver fibrosis, HSCs are the primary cell type in the liver responsible for excess collagen synthesis during hepatic fibrosis^16–18^. Following liver injury, HSCs undergo a complex transformation or activation process, changing from quiescent, vitamin A-storing cells to activated, myofibroblast-like cells. A large amount of extracellular matrix is then produced by fibroblasts and leads to liver fibrosis. The appearance of the cytoskeletal protein α-SMA is a marker of HSC activity^19^. In the present study, we found that ENMD-1068 inhibits the development of liver fibrosis in a fibrotic mouse model by diminishing α-SMA expression and collagen production. Although increased serum levels of ALT and AST were observed in mice treated with CCl_4_ alone, these elevations were reduced in the ENMD-1068-treated CCl_4_ groups. In agreement with our results, Knight *et al*. observed that the progression of liver fibrosis, hepatic collagen gene expression, and hydroxyproline content were reduced in PAR-2 deficient mice with liver fibrosis^8^. Thus, a reduction in α-SMA and collagen expression and ALT/AST levels observed in fibrotic liver tissues in ENMD-1068-treated mice may partially explain the decreased development of liver fibrosis in these mice. Furthermore, the safety and selectivity of ENMD-1068 has been demonstrated^10, 11, 20^ as no mice from the ENMD-1068 50 mg/kg treatment group were observed to have any obvious pathological changes in the abdomen. However, further investigation of the toxicity of ENMD-1068 is warranted.

Collagen gene expression is controlled by TGF-β1 and Smad family activation in tissues. TGF-β1 is a multifunctional cytokine that plays a crucial role in the regulation of cell growth, differentiation, and biosynthesis of extracellular connective tissue^21, 22^. TGF-β1 transduces signal from the membrane to the nucleus through transmembrane type I and II receptors by inducing phosphorylation of specific serine residues of the receptors. Among all Smad family members, Smad2/3 is closely associated with collagen gene expression. To evaluate whether PAR-2 is involved in collagen production, we investigated the expression of type I and III collagen and C-terminal phosphorylation of Smad2 influenced by TGF-β1 with or without ENMD-1068 treatment of isolated primary mouse HSCs. Interestingly, we found that the expression levels of type I and III collagen and TGF-β1 signaling-related phosphorylation of Smad2/3 C-terminal were significantly decreased in the ENMD-1068 treated HSCs. In agreement with our results, Chung *et al*. revealed PAR-2 synergizes with the TGF-β1 signaling pathway to contribute to renal injury and fibrosis^23^. Recently, Zeeh *et al*. reported that depleting pancreatic ductal adenocarcinoma (PDAC) and non-PDAC cells of PAR-2 by RNA interference strongly decreased TGF-β1-induced activation of Smad2/3 and Smad dependent transcriptional activity *in vitro* ^24^. Mussbach *et al*. also revealed that hepatic stellate cell line LX2 was unable to respond to TGF-β1 stimulation with phosphorylation of Smad3 after transfected with a PAR-2 shRNA^25^. These results strongly suggest that a functional cooperation between the TGF-β receptor(s) and PAR-2 is required for activation of Smad and non-Smad signaling. Since Smad2/3 are direct substrates of ALK5 which is in turn phosphorylated by TβRII^26–28^, further research is needed to prove that PAR-2 could affect the expression or activity of either ALK5 or TβRII in HSCs.

In conclusion, we have demonstrated that inhibition of PAR-2 activation in mice chronically exposed to CCl_4_ leads to a significant reduction in hepatic fibrosis. The mechanism of this effect is likely to be through a reduction in signal crosstalk between PAR-2 and the TGF-β1 signaling pathway. These novel findings suggest that PAR-2 represents a potential therapeutic target for patients with chronic progressive liver fibrosis.
