# Author Correction: ENMD-1068 inhibits liver fibrosis through attenuation of TGF-β1/Smad2/3 signaling in mice

**DOI:** 10.1038/s41598-019-55877-2

**Published:** 2019-12-10

**Authors:** Quan Sun, Yan Wang, Jie Zhang, Jing Lu

**Affiliations:** Department of Laboratory Animal Science, School of Basic Medical Science, Capital Medical University, Beijing, 100069 China

Correction to: *Scientific Report* 10.1038/s41598-017-05190-7, published online 14 July 2017

This Article contains errors in Figure 5, where the photograph for panel C3 incorrectly represents the group of ENMD-1068 25 mg/kg. The correct Figure 5 appears below.

**Figure 5:**
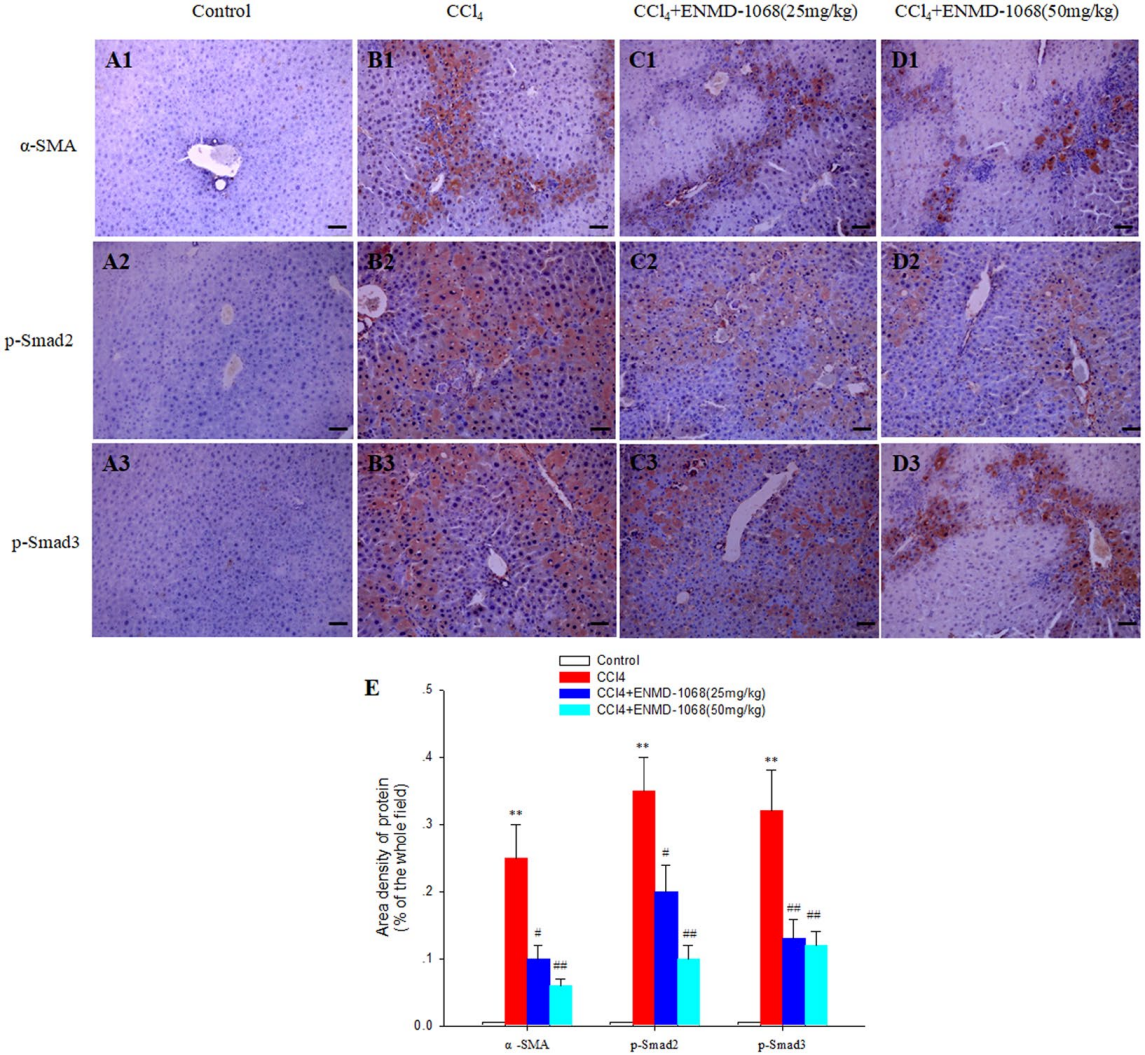
.

